# MicroRNAs in the Pathogenesis of Nonalcoholic Fatty Liver Disease

**DOI:** 10.7150/ijbs.59588

**Published:** 2021-04-29

**Authors:** Zhiqiang Fang, Guorui Dou, Lin Wang

**Affiliations:** 1 Department of Hepatobiliary Surgery, Xi-Jing Hospital, Fourth Military Medical University, Xi'an 710032, China.; 2 Department of Ophthalmology, Xi-Jing Hospital, Fourth Military Medical University, Xi'an 710032, China.

**Keywords:** miRNAs, Nonalcoholic fatty liver disease, NAFLD, Pathogenesis

## Abstract

Nonalcoholic fatty liver disease (NAFLD), or, more accurately, metabolic associated fatty liver disease, accounts for a large proportion of chronic liver disorders worldwide and is closely associated with other conditions such as cardiovascular disease, obesity, and type 2 diabetes mellitus. NAFLD ranges from simple steatosis to nonalcoholic steatohepatitis (NASH) and can progress to cirrhosis and, eventually, also hepatocellular carcinoma. The morbidity and mortality associated with NAFLD are increasing rapidly year on year. Consequently, there is an urgent need to understand the etiology and pathogenesis of NAFLD and identify effective therapeutic targets. MicroRNAs (miRNAs), important epigenetic factors, have recently been proposed to participate in NAFLD pathogenesis. Here, we review the roles of miRNAs in lipid metabolism, inflammation, apoptosis, fibrosis, hepatic stellate cell activation, insulin resistance, and oxidative stress, key factors that contribute to the occurrence and progression of NAFLD. Additionally, we summarize the role of miRNA-enriched extracellular vesicles in NAFLD. These miRNAs may comprise suitable therapeutic targets for the treatment of this condition.

## Introduction

Nonalcoholic fatty liver disease (NAFLD) is a clinicopathological syndrome caused by excessive fat deposition in hepatocytes of people who consume little or no alcohol. Due to dramatic lifestyle changes over recent decades, the number of NAFLD patients has increased significantly, accounting for a growing proportion of end-stage liver diseases 1. It is estimated that approximately a quarter of the world's population has NAFLD, but its global distribution is not uniform 2. NAFLD includes a collection of histopathological changes varying from simple steatosis to nonalcoholic steatohepatitis that can progress to cirrhosis and, eventually, hepatocellular carcinoma (HCC) 3.

Although the pathogenesis of NAFLD is very complex, existing evidence indicates that it is associated with the gut microbiome, bile acids, immunity, adipokines, oxidative stress, and genetic and epigenetic factors. Among the latter, miRNAs are suggested to play a key role in the occurrence and development of NAFLD. MiRNAs comprise at most 22 nucleotides and exert their activity by interfering with target mRNAs 4. They are indispensable for numerous biological processes, such as proliferation, apoptosis, development, differentiation, metabolism, and carcinogenesis 5. Evidence accumulated over recent years has indicated that miRNA dysregulation is involved in the occurrence and development of NAFLD, mainly through its effects on lipid metabolism, inflammation, apoptosis, fibrosis, insulin resistance, and oxidative stress. Recently, miRNA-enriched extracellular vesicles (EVs) have also been implicated in the pathogenesis of this condition. Accordingly, in this review, we focus on the role of miRNAs in the above-mentioned mechanisms, aiming to increase our understanding of the complex role of specific miRNAs in NAFLD and provide promising perspectives for therapeutic strategies to treat this disease.

## The role of miRNAs in regulating lipid metabolism in NAFLD

The liver is an important organ for lipid metabolism. Once lipid metabolic homeostasis is disrupted, excessive fat will accumulate in the liver, mainly in hepatocytes, which may eventually lead to the development of NAFLD. Lipid metabolism is influenced by several biological processes, but mainly including *de novo* lipogenesis (DNL), fatty acid intake, fatty acid oxidation (FAO), and very-low-density lipoprotein (VLDL) export. Once some of the above processes become dysregulated, hepatic lipid metabolism will be disrupted, manifesting as excessive hepatic triglyceride (TG) accumulation 6. Excessive TG deposition leads to the generation of hepatic steatosis, rendering the liver vulnerable to attack by factors such as proinflammatory cytokines, mitochondrial dysfunction, oxidative or endoplasmic reticulum (ER) stress, and the gut microbiome, further leading to the occurrence of inflammation, cellular apoptosis, or necrosis and fibrosis 7.

MiR-21, widely expressed in many types of tissues in humans 8, is dysregulated in cancer, inflammation, fibrosis, and NAFLD 9. The level of circulating miR-21 and its expression in the liver is elevated in NAFLD patients and mouse models 9-11. Calo et al. found that liver-specific miR-21/miR-21 knockout (LImiR21KO) mice fed a high-fat diet exhibited decreased hepatic steatosis through the regulation of several key transcription factors such as FOXA2, FOXO1, HNF4α, STAT3, and INSIG2 12. Moreover, miR-21 was also shown to inactivate the WNT/β-catenin signaling pathway by targeting LRP6, thereby aggravating lipid accumulation and inflammation 13, while Huang et al. reported that long noncoding RNA (lncRNA) MEG3 competitively bound to miR-21 with LRP6 and promoted lipid accumulation through inhibiting the mTOR pathway 14. MiR-21 also promotes hepatosteatosis and cancer progression through the Hbp1-p53-Srebp1c pathway 15. Several studies have found that inhibiting miR-21 could alleviate steatosis through activating PPARα 16, 17; however, the role of miR-21 in targeting PPARα requires further investigation 18. In addition, miR-21 also promotes the progression of NAFLD-related HCC (NAHCC) through PI3K/AKT, TGF-β, and STAT3 signaling 19, suggesting that miR-21 may be involved in various stages of NAFLD, from steatosis to NAHCC.

MiR-122, the most abundant miRNA in the liver 20, is expressed specifically in hepatocytes 8 and is involved in NAFLD development mainly through regulating lipid metabolism 21. In NASH patients, the level of circulating miR-122 is significantly upregulated 22 and the hepatic expression of miR-122 is decreased 23, indicating that the miR-122 present in serum is released by hepatocytes 24. MiR-122 can regulate lipid metabolism via multiple signaling pathways. Chai et al. found that free fatty acids (FFAs) could induce the expression of miR-122 and promote its secretion to circulating blood, and that miR-122 could suppress TG synthesis by targeting Agpat1 and Dgat1 as well as increasing beta-oxidation. The authors also found that inhibiting miR-122 led to the downregulation of FoxO1 and the upregulation of PPARγ, indicating that miR-122 is involved in multiple lipid metabolism-related pathways 25. Intriguingly, adipose can secrete miR-122-containing exosomes that are absorbed by the liver, thereby alleviating NAFLD progression, which partly accounts for the paradoxically high levels of circulating miR-122 and its low expression in the liver of NAFLD patients 26. Additionally, miR-122 can indirectly downregulate FASN and ACC and decrease cholesterol synthesis to ameliorate steatosis 27. In addition to their function as upstream regulators of target mRNAs, microRNAs can themselves serve as downstream targets. For example, circRNA_002581 can suppress miR-122 expression, leading to the upregulation of CPEB1 and phosphorylated-mTOR and the concomitant suppression of autophagy, finally resulting in aggravated steatosis, inflammation, hepatocyte apoptosis, and oxidative stress 28. In contrast, however, Long et al. reported that miR-122 could directly bind to the 3'-UTR of Sirt1, thereby inhibiting its expression and promoting lipogenesis 29. These contradictory results suggest that the function of miR-122 in NAFLD pathogenesis is complex and warrants further investigation.

Although the expression of miR-34a in hepatocytes is not high, miR-34a may nonetheless be closely correlated with lipid metabolism. It is well known that Sirt1 is a direct target of miR-34a. Ding et al. demonstrated that inhibiting miR-34a could alleviate hepatosteatosis by targeting PPARα and Sirt1 30. Cholesterol metabolism also influences NAFLD severity. By inhibiting Sirt1 expression, miR-34a can promote that of HMGCR, which leads to increased free cholesterol levels and the aggravation of NAFLD 31. Moreover, miR-34a decreases VLDL secretion by downregulating the expression of HNF4α, leading to increased lipid accumulation 32. In addition to functioning as an upstream regulator, miR-34a is itself targeted and upregulated by FoxO3, which promotes palmitate (PA)-induced cholangiocyte lipoapoptosis 33. Additionally, circRNA_0046367 releases the inhibitory effect of miR-34a on PPARα through disrupting the miRNA/mRNA interaction, thus alleviating lipid peroxidation 34. Kim et al. identified the inhibitory role of LXRα in hepatocyte autophagy by upregulating the expression of miR-34a 35, while the farnesoid X receptor/miR-34a/sirtuin 1 pathway was proposed as a potential entry point for treating NAFLD 36. MiR-33 also plays a pivotal role in lipid metabolism in NAFLD as excellently illustrated by Rottiers and Näär 37. Other miRNAs with lipid metabolism regulatory roles in NAFLD are summarized in Table 1.

In summary, this part mainly focused on the regulatory mechanisms underlying the roles of several miRNAs in lipid metabolism in NAFLD/NASH and briefly introduced their expression levels in peripheral blood and liver. Given that serum levels of some miRNAs, such as miR-21, miR-122, and miR-192, differ significantly between NAFLD/NASH patients and healthy controls, they have the potential to serve as biomarkers for noninvasive diagnosis. MiRNAs participate in every stage of lipid metabolism, including DNL, fatty acid oxidation, lipid transportation, and cholesterol metabolism. Lipid accumulation is regarded as the “first hit” in NAFLD. Consequently, alleviating steatosis represents an efficient strategy for blocking NAFLD progression and the above-mentioned miRNAs may provide a theoretical basis for the miRNA-based treatment of this disease.

## MiRNAs orchestrate the progression of NAFLD via insulin signaling

Insulin resistance (IR) refers to a decrease in the efficiency of insulin in promoting glucose uptake and utilization. To compensate for increased blood glucose levels, the body produces and secretes excessive amounts of insulin, known as hyperinsulinemia 57. Numerous studies have suggested that both genetic and environmental factors are related to the occurrence of IR, with obesity and hyperglycemia playing a key role 58. Insulin resistance plays a critical role in the pathogenesis of NAFLD, increasing DNL and accelerating adipose tissue lipolysis, thereby leading to excessive fatty acid accumulation in the liver 57. IR also disrupts the function of adipose tissue, consequently disrupting the normal regulation of inflammatory cytokines and adipokines 59. The above consequences result in inflammation, oxidative stress, and apoptosis, which in turn maintain and promote the IR condition 58.

Numerous miRNAs have been shown to participate in insulin signal transduction and may be therapeutic targets in NAFLD. For instance, the PI3K/PDK1/AKT pathway is a key player in insulin signaling, and PI3K activity is regulated by phosphatase and tensin homolog (PTEN) and Src homology 2 domain-containing inositol 5'-phosphatase 2 (SHIP2). The hepatic expression of miR-152 was markedly downregulated in *db*/*db* mice and mice fed a high-fat diet, which leads to impaired hepatic glycogenesis—a hallmark of IR in hepatocytes—*via* PTEN upregulation 60; meanwhile, miR-499-5p exerts a similar effect *via* the same target 61. This results in dysregulated insulin signaling and excessive fatty acid accumulation in the liver. Several other miRNAs are known to target insulin signaling pathways related to the pathogenesis of NAFLD. For instance, Fu et al. showed that the downregulation of miR-26a contributed to the development of IR *via* multiple pathways 62, while Wang and colleagues reported that high miR-497 levels could inhibit insulin receptor expression and inactivate the IRS-1/PI3K/Akt/GSK-3b/GS pathway, thereby inducing hepatic IR 63. MiR-15b exerts the same effect *via* InsR 64. MiR-30b was upregulated in the liver of rats fed a high-fat diet and its overexpression can promote IR through suppressing sarco(endo)plasmic reticulum Ca^2+^-ATPase 2b (SERCA2b) translation 65. Moreover, miR-206 can simultaneously facilitate insulin signaling and decrease hepatic lipogenesis through inhibiting protein tyrosine phosphatase non-receptor type 1 (PTPN1) 66. In contrast, Xu et al. reported that miR-190b had the opposite effect on lipid metabolism and insulin sensitivity in NAFLD 67. In addition, the serum level of miR-103 is higher in NAFLD patients than in healthy controls and is closely related to IR, and may serve as a biomarker for this condition in NAFLD 68. The brief information of the above-mentioned miRNAs has been summarized in **Table 2**.

In summary, we briefly introduced several miRNAs involved in the regulation of insulin signaling and, consequently, lipid metabolism. It is well known that IR contributes to excessive lipid accumulation in the liver, which can lead to steatosis and the progression from NAFLD to NASH. Moreover, therapy focusing on insulin signaling may also be beneficial for other metabolic disorders such as T2DM and obesity, as well as for NAFLD patients with these conditions.

## The role of miRNAs in modulation of oxidative stress and ER stress in NAFLD

Redox homeostasis is vital for the maintenance of normal cellular functions and cell fate commitment 70. Oxidative stress, which refers to disturbances in the regulatory role of reactive oxygen species (ROS), can interfere with various physiological processes and participate in the pathogenesis of various diseases 71. ROS are widely defined as reactive oxidizing substances generated mainly in mitochondria, peroxisomes, and ER as a result of both enzymatic and nonenzymatic reactions 70. Excessive ROS production disrupts normal biological processes by damaging molecules such as DNA, proteins, and lipids 70. The ER is associated with protein maturation. When excessive FFAs flow into the liver, the ER in hepatocytes must process increased amounts of protein, leading to greater numbers of misfolded proteins, defined as ER stress, and the activation of a protective program called the unfolded protein response (UPR). Sustained activation of the UPR promotes NAFLD progression by affecting lipid accumulation, mitochondrial activities, and insulin signaling 72. During NAFLD progression, an excess of FFAs leads to ROS overproduction, which can damage mitochondria and lead to lipotoxicity 73. In turn, lipotoxicity leads to ER stress, increased inflammation, hepatocyte damage, and, finally, hepatocyte death 74. Interestingly, ER stress also promotes ROS production 74. Therefore, oxidative stress and ER stress can interact with each other and jointly promote NAFLD development.

In addition to IR, miR-26a also plays a role in ER stress. Xu et al. found that miR-26a was induced in liver cell lines treated with an ER stress inducer while hepatic expression of miR-34a is reduced in NAFLD patients, suggesting that there is an ER stress/miR-26a feedback circuit in hepatocytes. In other words, ER stress in NAFLD stimulates the upregulation of miR-26a, which, in turn, alleviates ER stress and lipid accumulation 75. Cheng and colleagues proposed that miR-421 inhibited SIRT3, thereby interfering with normal mitochondrial function in NAFLD. In a mouse model of NAFLD, miR-421 expression was markedly increased, which aggravated oxidative stress and increased lipid accumulation through SIRT3/FOXO3 signaling. The authors concluded that suppressing hepatic miR-421 might alleviate oxidative stress-induced cellular damage in NAFLD 76. Nuclear factor-erythroid 2-related factor 2 (Nrf2), a key modulator of the cellular antioxidant system, can also affect the pathogenesis of NAFLD. The expression of Nrf2 can be inhibited when miR-27a is overexpressed, leading to increased ROS production and lipid accumulation 41. Furthermore, miR-136 was reportedly downregulated by high-content hydrogen water, resulting in increased levels of Nrf2 and MEG3 77. Cytochrome C participates in ROS production and mitochondrial apoptosis. Zhang et al. found that the upregulation of miR-96-5p ameliorated NAFLD through inhibition of the p66shc/cytochrome C cascade 78. The brief information of the above-mentioned miRNAs has been summarized in **Table 3**.

In summary, we briefly described the roles of several miRNAs in the regulation of oxidative stress and ER stress. Oxidative stress and ER stress contribute to lipid accumulation and hepatocyte apoptosis, thus aggravating the progression of NAFLD. Many miRNAs, such as miR-26a, miR-27a, and miR-136, can improve the metabolic state of liver cells by regulating key redox pathways and molecules, thereby affecting NAFLD progression. These findings may provide clues for aiding the monitoring of disease progression and the development of miRNA-based targeted therapy for the treatment of NAFLD.

## MiRNAs participate in the regulation of NADLD related hepatocyte apoptosis

Apoptosis is common in acute and chronic liver injury. Different from necrosis, apoptosis is executed through a precisely designed program that usually occurs in only a single cell, having little effect on surrounding cells such that tissue homeostasis or organ development is not disrupted 79. Under physiological conditions, cellular apoptosis in the liver is relatively rare and well-organized. However, inflammation, lipotoxicity, mitochondrial dysfunction, or other injuries can result in excessive apoptosis in the liver. Several key molecular pathways, such as the Bcl-2, caspase, and c-Jun N-terminal kinase (JNK) signaling pathways, are involved in the regulation of hepatocyte apoptosis, a key factor in the pathogenesis of NAFLD/NASH. Apoptotic hepatocytes will, in turn, stimulate surrounding HSCs and immune cells, leading to aggravated hepatic inflammation and the occurrence and progression of fibrosis 80.

In addition to playing a role in hepatic inflammation and fibrosis, miR-223 is also involved in hepatocyte apoptosis. For instance, Qadir et al. found that knockdown of miR-223 could alleviate Fas-induced hepatocyte apoptosis *via* targeting IGF-1R 81, suggesting that miR-223 may also interfere with the progression of NAFLD through regulation of hepatocyte apoptosis. This possibility merits further investigation. Additionally, Feng et al. found that miR-24 could suppress hepatocyte apoptosis *via* the proapoptotic Bcl-2 homolog, BIM, a contributor to TNF-α-induced apoptosis, in a lipopolysaccharide (LPS)-induced mouse model of acute liver failure 82; however, whether miR-24 exerts the same anti-apoptotic function in NAFLD remains unknown. Deoxycholic acid (DCA) is a free bile acid reported to induce apoptosis *via* multiple pathways 83. Rodrigues et al. showed that DCA inhibited the NF-κB pathway, decreased the expression of miR-21, increased the expression of the miR-21 proapoptotic target programmed cell death 4 (PDCD4), and promoted apoptosis in primary rat hepatocytes. In contrast, the overexpression of miR-21 decreased the levels of hepatocellular apoptosis 83. The same authors found that DCA activated miR-34a to induce apoptosis *via* targeting SIRT1 84 and other groups have also reported on the proapoptotic role of miR-34a 85,86. The BH3-only protein PUMA is upregulated during lipoapoptosis. Cazanave et al. showed that the liver expression of miR-296 was decreased in NASH patients, while the overexpression of miR-296 in Huh7 cells ameliorated lipoapoptosis *via* targeting PUMA 87. The brief information of the above-mentioned miRNAs has been summarized in **Table 4**.

In summary, we introduced several miRNAs involved in the regulation of hepatocyte apoptosis, mainly focusing on the associated mechanisms. Apoptotic hepatocytes can contribute to the progression of inflammation and fibrosis, indicating that hepatocyte apoptosis plays a central role in the pathogenesis of NAFLD and NASH. Alleviating hepatocyte apoptosis will likely limit inflammation and fibrosis, thereby inhibiting the progression of NAFLD. This observation highlights that miRNA-based targeted therapy for inhibiting hepatocyte apoptosis holds great promise for the treatment of NAFLD/NASH. Nevertheless, most studies investigating hepatocellular apoptosis-related miRNAs have focused on HCC; other miRNAs associated with hepatocellular apoptosis in NAFLD/NASH need to be identified and explored.

## The role of miRNAs in NAFLD related hepatic inflammation

Inflammation participates in the pathogenesis of many liver diseases, including NAFLD 91. It is a required pathological feature for defining NASH and differentiating NASH from simple steatosis 92. Inflammation is also a key factor in the progression of NASH to cirrhosis and, finally, HCC 93. Inflammation plays both injurious and beneficial roles in the pathogenesis of NAFLD, depending on the types of inflammatory cells and inflammatory mediators involved, as well as on the stage of the disease 94. Inflammation can help heal the liver by promoting liver regeneration and the clearing of dead or unhealthy cells. In contrast, continuous inflammation can negatively affect normal physiological activities and destroy normal tissue structure, and can, in the end, lead to the occurrence of fibrosis. Hepatic inflammation in NAFLD likely originates outside the liver and inside the liver, a topic that has been reviewed elsewhere 95. MiRNAs have been shown to participate in hepatic inflammatory responses and influence the progression of NAFLD/NASH 96, 97.

Until recently, miR-223 expression was thought to be neutrophil/myeloid-specific 8, however, it is now known that miR-223 is also expressed in hepatocytes 98. MiR-223, an miRNA with anti-inflammatory functions, was reported to ameliorate hepatic inflammation in NASH *via* targeting Cxcl10 and transcriptional coactivator with PDZ-binding motif (TAZ) in hepatocytes 89. Moreover, miR-223 plays a vital role in the crosstalk between inflammatory cells and hepatocytes *via* EVs. For example, IL-6 reportedly stimulates myeloid cells to release exosomes containing miR-223 that are absorbed by hepatocytes. Subsequently, miR-223 alleviates liver fibrosis by suppressing the expression of its profibrotic target genes, including Nlrp3, Igf1r, Cxcl10, and TAZ 88. Additionally, hepatocytes can absorb neutrophil-derived miR-223-enriched EVs in a LDLR- and apolipoprotein E (APOE)-dependent manner 99. The specific role of these two molecules in the selective control of miRNA-223 transfer has potential as a therapeutic target for the treatment of NASH.

MiR-194 is also involved in NAFLD-related inflammation, but not in HCC progression and metastasis 8. Nie et al. demonstrated that miR-194 could suppress FXR/Nr1h4 expression, thereby promoting inflammatory responses and metabolic dysfunction 100, while Tian et al. showed that the overexpression of miR-194 could reduce the expression levels of TRAF6, a downstream effector of the TLR4 pathway, thus alleviating inflammatory responses 101. These contradictory results illustrate the complexity associated with miRNA regulatory networks and that of the function of a single miRNA. Additionally, several miRNAs involved in the regulation of lipid metabolism also play a role in inflammation. For instance, exosomal miR-192-5p derived from NAFLD hepatocytes could promote macrophage activation through the Rictor/Akt/FoxO1pathway 102. This suggests that hepatocyte-derived exosomes can also have an important effect on surrounding inflammatory cells. Besides increasing lipid accumulation, miR-378 can also promote hepatic inflammation through activating the NF-κB/TNF-α pathway 103. Similarly, miR-125b can also enhance NF-κB signaling by targeting TNFAIP3, thereby aggravating inflammatory responses 48. Further, miR-451 activates the AMPK/AKT pathway, thus decreasing hepatic inflammation by suppressing the activation of NF-κB *via* targeting Cab39 104. The brief information of the above-mentioned miRNAs has been summarized in **Table 5**.

In summary, we introduced the specific mechanisms underlying the roles of several miRNAs involved in the regulation of hepatic inflammation in NASH. Several canonical inflammation-related pathways, such as the NF-κB/TNF-α and TLR4 pathways, are regulated by miRNAs, including miR-194, miR-378, miR-125b, and miR-451. MiR-223 can alleviate hepatic inflammation and fibrosis *via* multiple targets. The crosstalk between hepatocytes and surrounding cells, such as macrophages and neutrophils, plays a pivotal role in hepatic inflammation. It is increasingly recognized that EVs are involved in the occurrence and development of hepatic inflammation in NASH. The function of miR-223-enriched EVs in inflammation has been excellently illustrated by Wang et al. 88 and He et al. 99.

## MiRNAs influence NAFLD by regulating the progression of liver fibrosis

Liver fibrosis is a pathological process in which extracellular matrix accumulates and damage repair persists 106. Different stages of fibrosis indicate different outcomes in NAFLD and fibrosis is an important predictor of mortality 107. Hepatocyte lipoapoptosis is the main driving force for the progression of fibrosis 108. Pathological conditions promote the activation of a large number of HSCs, which are the main source of fibrogenic myofibroblasts (MFs; primary cells that produce extracellular matrix). An excessive number of MFs leads to massive EM deposition, the main characteristic of liver fibrosis 107. Multiple signal transduction pathways can regulate HSC activation, including the TGF-β/Smad, Notch, Wnt, Hedgehog, and integrin pathways. MiRNAs are currently considered to be a turning point for the noninvasive diagnosis of NAFLD 109. Given that many miRNAs are involved in the development of fibrosis and HSC activation, they may provide references for the treatment of NAFLD/NASH and the prevention of disease progression.

Using high-throughput sequencing, Leti et al. found that the levels of many miRNAs were altered in NAFLD-related fibrosis, thereby providing new insights into the mechanisms underlying the pathogenesis of this condition 110. Dattaroy et al. showed that the leptin-NADPH oxidase-mediated induction of miR-21, *via* the TGF-β signaling pathway, was a key regulatory step in NASH-related fibrogenesis 111. In addition to promoting hepatosteatosis, miR-21 also contributes to liver fibrosis by inhibiting PPARα signaling 16. High miR-21 expression is mainly observed in bile duct cells and inflammatory cells in NASH patients and mouse models 16, suggesting that suppressing hepatic miR-21 expression may reduce fibrosis by suppressing inflammation. It has been proposed that miR-21 may serve as a plasma biomarker for fibrotic liver disease 112. Intriguingly, miR-21 was reported to be unnecessary for HSC activation and the development of liver fibrosis 113. Consequently, the role of miR-21 in liver fibrosis and HSC activation remains unclear and requires further investigation. MiR-122 is highly expressed in hepatocytes and is correlated with cholesterol metabolism 114. Csak et al. reported that the overexpression of miR-122 alleviated liver fibrosis by targeting HIF-1α, vimentin, and MAP3K3 114. Moreover, Du et al. showed that the proliferation and activation of HSCs could be suppressed by miR-146a-5p in NASH *via* Wnt1 and Wnt5a 115.

The Wnt signaling pathway is involved in various biological processes, including apoptosis, and the canonical Wnt/β-catenin signaling pathway is one of the main inducers of HSC apoptosis 116,117. MiR-214 is markedly upregulated during HSC activation, which results in the inhibition of the expression of suppressor-of-fused homolog (Sufu), a negative regulator of the Hedgehog pathway, and the consequent promotion of HSC activation and liver fibrosis 118. Moreover, miR-214 also enhances the activity of the EGFR and TGF-β signaling pathways by targeting Mig6, a negative regulator of EGFR signaling, thereby promoting HSC activation and liver fibrosis 119. However, the role of miR-214 in NASH-related fibrosis remains unclear. Wang et al. demonstrated that miR-130a-3p targeted TGFBR1 and TGFBR2, which inactivated HSCs, promoted their apoptosis, and ameliorated fibrosis in NASH 120. The TGF-β/Smad pathway can block the progression of liver fibrosis by inhibiting HSC collagen secretion and cell activation. Using a CCL4-induced liver fibrosis mouse model, Tsay et al. showed that downregulating miR-221-3p in hepatocytes could mitigate the symptoms of liver fibrosis by suppressing HSC activation *via* G protein alpha inhibiting activity polypeptide 2 (GNAI2) 121. MiR-29 can also decrease inflammation and fibrosis in NASH by suppressing CD36 43. The brief information of the above-mentioned miRNAs has been summarized in **Table 6**.

In this section, we summarized the function of some miRNAs involved in the regulation of liver fibrosis. Many miRNAs participate in the regulation of classical fibrosis-related signaling pathways such as the TGF-β/Smad, Wnt, and Hedgehog pathways. A few miRNAs, including miR-214 and miR-221-3p, play a role in drug-induced fibrosis instead of NASH, and the role of these miRNAs in NASH-induced fibrosis needs to be investigated. Many miRNAs affect the progression of NAFLD through multiple processes, including steatosis, apoptosis, inflammation, and fibrosis. Consequently, whether these miRNAs directly interfere with the process of fibrosis or indirectly promote/aggravate fibrosis through affecting steatosis or inflammation needs to be determined. This will help clarify the underlying mechanisms and provide a solid theoretical basis for miRNA-based targeted therapy.

## Extracellular vesicles (EVs) in NAFLD: potential biomarkers and therapeutic targets

EVs refer to vesicles encapsulated by phospholipid bilayers that are released by various types of cells. They can be classified into three groups, namely, exosomes (40-150 nm in diameter), microvesicles (50-1,000 nm in diameter), and apoptotic bodies (500 nm in diameter), according to their cellular origins 122. EVs can carry numerous particles, including enzymes, growth factors, proteins, lipids, and noncoding RNAs, thereby mediating cell-to-cell communication 123. Over recent years, EVs have been found to contribute to various liver diseases, such as hepatitis B and C, alcoholic liver disease (ALD), NAFLD, and HCC. Given that EVs carry specific proteins and lipids, they can recognize specific target cells through receptor-mediated or membrane-mediated processes, which also allow EVs to serve as biomarkers and therapeutic targets 122.

Several studies have suggested that circulating EV levels are increased in both experimental and human NASH 124. Studies investigating the functions of EVs secreted by hepatocytes have mostly focused on their effects on liver cells, and EVs have been found to be mainly correlated with the regulation of inflammation and fibrosis. For example, in a NASH mouse model, hepatocytes released integrin β1-enriched EVs, which was absorbed by monocytes and increased their adhesion to liver sinusoidal endothelial cells, thus aggravating inflammation and fibrosis 125. Jiang et al. showed that lipotoxic hepatocyte-derived and miR-1-enriched EVs promoted endothelial inflammation and atherogenesis, which was proposed to provide a theoretical basis for the treatment of atherosclerosis patients with NAFLD 126. Lipotoxic hepatocyte-derived EVs can also be internalized by HSCs and induce their activation, likely through the downregulation of PPARγ induced by miR-128-3P contained in the EVs 127. Hepatocyte-derived EVs can also be internalized by macrophages. For instance, sphingosine 1-phosphate (S1P)-enriched EVs released by lipotoxic hepatocytes can induce macrophage chemotaxis 128. Similarly, mixed lineage kinase 3 (MLK3) is induced in lipotoxic hepatocytes, leading to the release of EVs containing C-X-C motif ligand 10 (CXCL10) and the induction of macrophage chemotaxis 129. Endoplasmic reticulum to nucleus signaling 1 (ERN1) is also induced in hepatocytes of NASH livers and promotes EV release and macrophage chemotaxis 130. Additionally, lipid stimulates death receptor 5 (DR5) and leads to increased release of hepatocyte-derived EVs containing TNF-related apoptosis-inducing ligand (TRAIL) and the upregulation of IL-1β and IL-6 expression in macrophages 131. Furthermore, lipotoxic hepatocyte-derived exosomal miR-192-5p activates macrophages and increases the production of proinflammatory cytokines 102. Inflammatory cells also release EVs to interact with hepatocytes. For example, IL-6 induces macrophages to release miR-223-enriched EVs, which are internalized by hepatocytes and lead to a reduction in the expression of profibrotic TAZ 88. Neutrophils can also release miR-223-enriched EVs and ameliorate hepatic inflammation and fibrosis 99. Interestingly, EVs can also be transferred among cells of the same type. For instance, miR-214-enriched exosomes can be transferred between HSCs, thereby reducing fibrogenesis 132. Recent studies have demonstrated that adipose tissue-derived EVs can be transferred into the liver, such as that seen for adipose tissue macrophage-derived exosomal miR-155, which impairs the insulin sensitivity of hepatocytes *via* targeting PPARγ 133.

Given that circulating miRNAs are transported *via* EVs and those specific proteins and EVs can be absorbed by specific cells, miRNA-enriched EVs have great potential to serve as effective biomarkers and therapeutic targets. Considering that miRNAs in EVs are more stable than those exposed to blood, the diagnostic value of detecting miRNAs in EVs may be greater than that of detecting miRNAs extracted from serum. However, due to the lack of a uniform standard for separating EVs from serum, the diagnostic potential of EVs is currently limited. Recently, a few laboratories have tried to use EVs containing specific miRNAs to affect NAFLD progression. For example, Li et al. delivered miR-199a-5p- or anti-miR-199a-5p-containing exosomes to respectively aggravate or ameliorate lipid accumulation in a NAFLD mouse model 134. However, the clinical translation of treatment based on miRNA-containing EVs is in its infancy and requires substantial investigation and confirmation.

**Figure 1:**
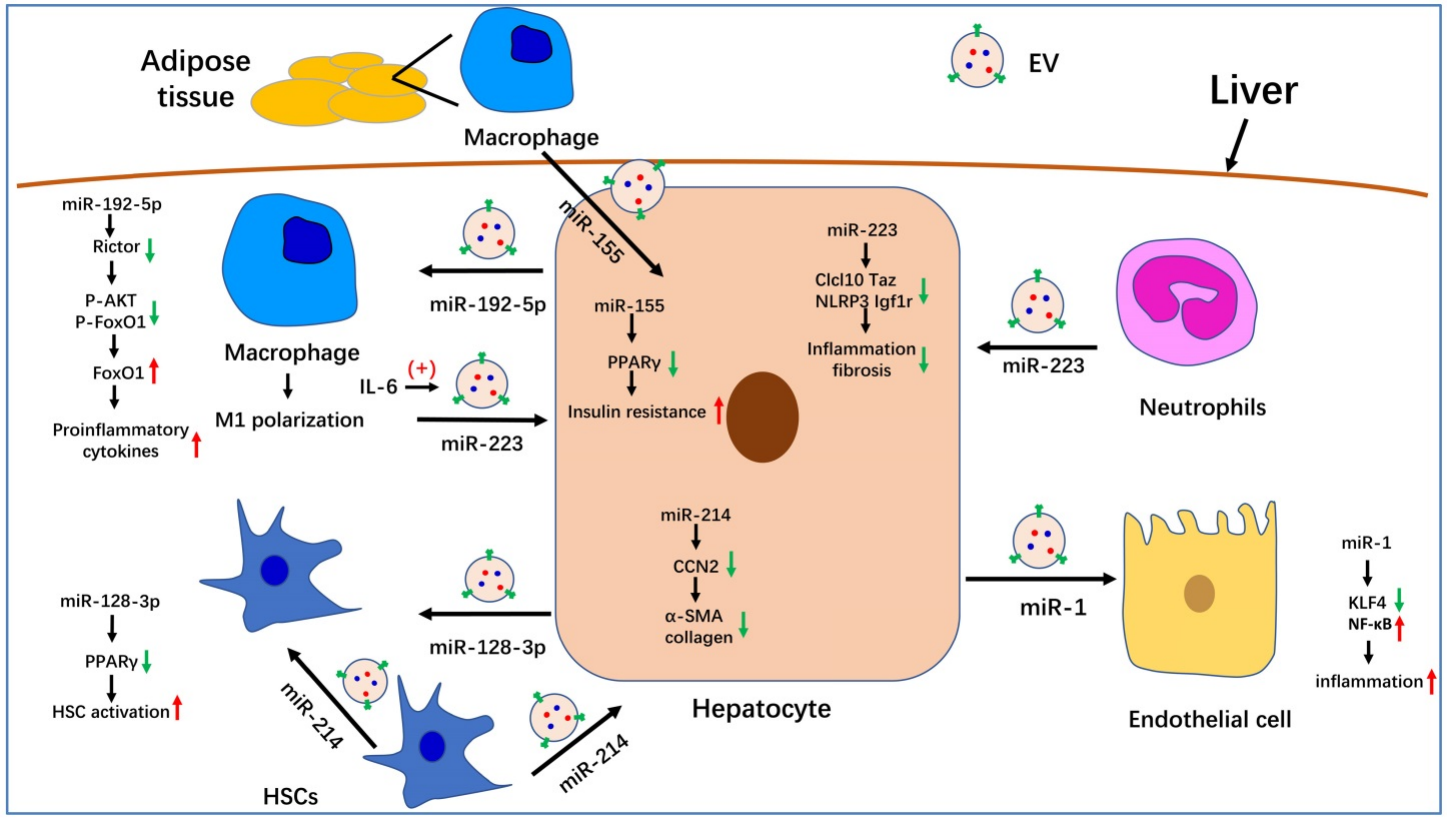
** The role of miRNA-enriched extracellular vesicles (EVs) in intercellular communication in nonalcoholic fatty liver disease (NAFLD).** Adipose tissue-derived macrophages secrete miR-155-enriched EVs, which are internalized by hepatocytes and impair insulin sensitivity. MiR-223-enriched EVs released by neutrophils and macrophages are absorbed by hepatocytes and improve hepatic inflammation and fibrosis. MiR-214-containing EVs encapsulated by hepatic stellate cells (HSCs) can simultaneously be absorbed by HSCs and hepatocytes and decrease the expression of fibrogenesis-related genes by suppressing CCN2. Hepatocytes also secrete miR-192-5p-enriched EVs, which are internalized by macrophages, where they promote inflammation and induce M1 polarization. Hepatocytes also release miR-1-enriched EVs, which are transferred to endothelial cells to promote inflammation by targeting KLF4. Moreover, miR-128-3p-containing EVs released by hepatocytes can be taken up by HSCs and increase HSC activation by inhibiting PPARγ.

In summary, we summarized the known roles of EVs in the pathogenesis of NAFLD. Serum EV levels are significantly increased in NASH mouse models and patients. Many molecules that are involved in NAFLD progression have been detected in EVs, especially miRNAs. The role of partial miRNA-enriched EVs in NAFLD has been summarized in Figure 1. Because EVs can be internalized by specific cells through specific membrane receptors, they function as a means of intercellular communication. These characteristics endow EVs with the potential to serve as biomarkers and therapeutic targets for the treatment of NAFLD/NASH.

## Conclusion

**Figure 2:**
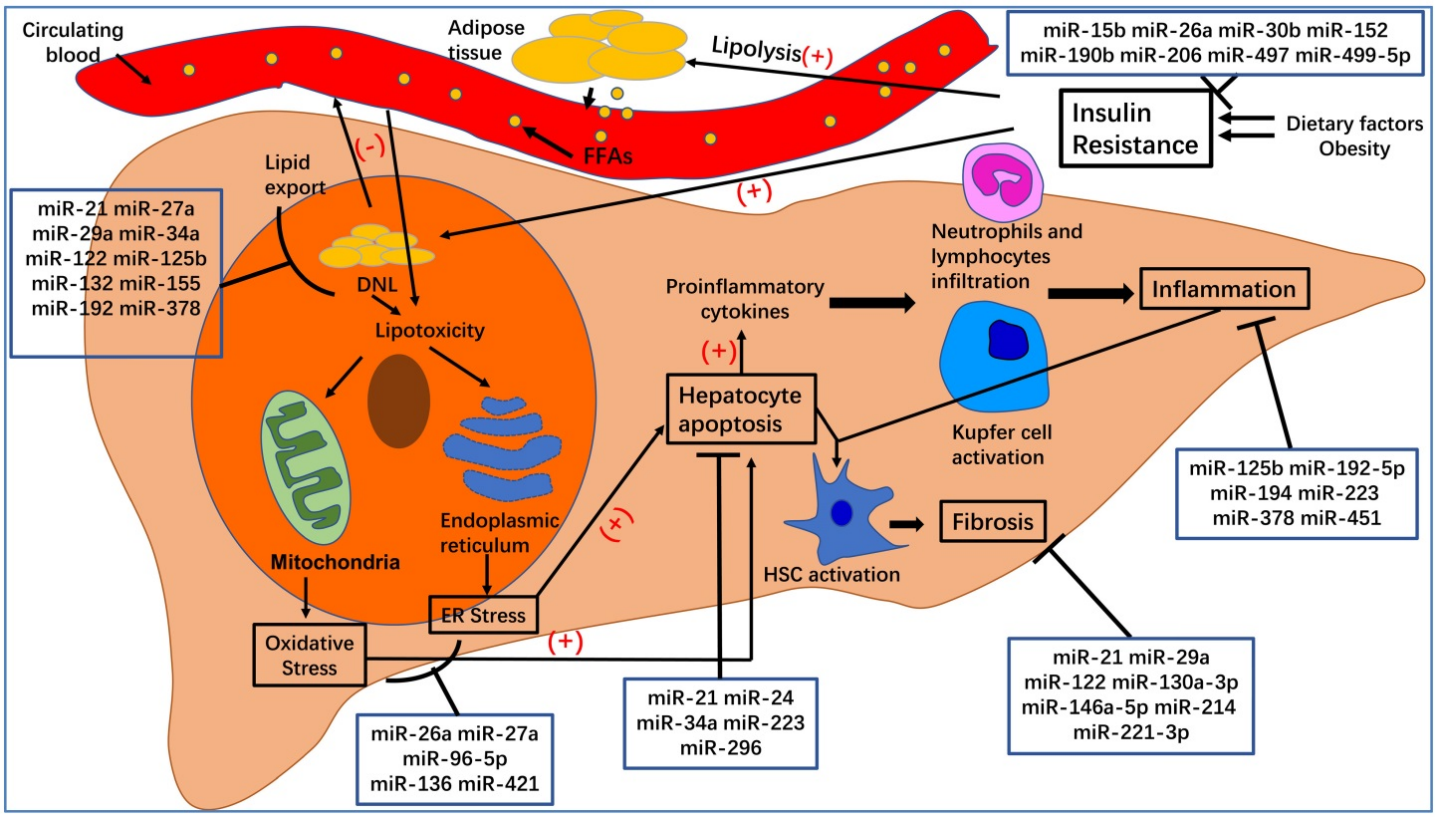
** The pathogenesis of nonalcoholic fatty liver disease (NAFLD) and nonalcoholic steatohepatitis (NASH).** Dietary factors and obesity, important for the development of insulin resistance, increase the lipolysis of adipose tissue and *de novo* lipogenesis (DNL) in hepatocytes. An excess of free fatty acids from circulating blood enters the liver, which, together with increased DNL in hepatocytes, leads to hepatocyte steatosis and lipotoxicity. The latter can affect the normal functioning of mitochondria and endoplasmic reticulum, leading to oxidative stress and endoplasmic reticulum stress, which can increase hepatocyte apoptosis. Apoptotic hepatocytes increase the production of proinflammatory cytokines, recruit inflammatory cells to the liver, activate Kupfer cells, and lead to inflammation, which is a hallmark of NASH. At the same time, inflammation and hepatocyte apoptosis promote the activation of hepatic stellate cells (HSCs), which will transform into myofibroblasts and produce extracellular matrix. Excessive deposition of extracellular matrix leads to the occurrence of liver fibrosis.

Here, we have summarized the roles of specific miRNAs in the pathogenesis of NAFLD (see Figure 2). We expected that this review may be of benefit to clinical research and disease treatment. MiRNAs undoubtedly play a critical role in the post-transcriptional regulation of target genes involved in the pathogenesis of NAFLD/NASH. Elaborating on the function of each miRNA in NAFLD is beyond the scope of this review. Instead, we have focused on several widely studied miRNAs, such as miR-21, miR-26a, miR-34a, miR-122, miR-194, and miR-223. Additionally, because exosomal miRNAs have attracted significant attention over recent years, we also summarized the role of EVs in NAFLD and the function of miRNA-enriched EVs in the pathogenesis of NAFLD. The relationship between miRNAs and their targets is complex. For example, miR-21 regulates NAFLD progression *via* multiple targets, including FoxA2, HNF4α, STAT3, 12 LRP6, 13 Hbp1, 15 PPARα, 16 SMAD7, 111 and PDCD4 83. One target gene can be regulated by several miRNAs. For instance, Sirt1 can be regulated by miR-34a, 30, 31, 36, 84-86 miR-122, 29 and miR-132 47. Moreover, multiple-to-multiple relationships between miRNAs and their target genes have been observed in HCC 135. This phenomenon indicates that the miRNA regulatory network in NAFLD is complex and warrants further investigation.

Because of their stability, detectability, and evident changes in circulating levels in health and disease, miRNAs can serve as efficient, noninvasive biomarkers. For example, serum levels of miR-21 10, 11, miR-122, 22 and miR-192 102 are markedly upregulated in NAFLD and could serve as serum biomarkers for the early diagnosis of this disease. Moreover, miR-34a, miR-122, and miR-192 may be suitable for use as biomarkers to distinguish NAFLD and NASH severity 8. However, it is difficult to diagnose NAFLD *via* a single miRNA because determining specificity remains problematic. For instance, the serum levels of miR-21 also increase in other liver diseases such as ALD, HCC, and viral hepatitis 136. It is therefore more effective to use a combination of miRNAs or a combination of miRNAs and traditional biomarkers for the noninvasive diagnosis of NAFLD. Accordingly, Liu et al. proposed a composite biomarker that included miR-21, miR-192, miR-505, and alanine aminotransferase (ALT) to improve noninvasive NASH diagnosis 11, while Thietart et al. showed that a cluster of 12 miRNAs in serum EVs could distinguish patients with NASH from those with chronic hepatitis B and C or healthy controls 137. Recently, an increasing number of studies have demonstrated that numerous miRNAs are transferred *via* EVs and that miRNAs in EVs are more stable than those in circulating blood. Therefore, isolating EVs from serum and detecting their miRNA concentrations may represent a novel and efficient strategy for noninvasive diagnosis. For instance, as mentioned above, exosomal miR-128-3p 127, miR-192-5p 102, miR-223, 88,99 and miR-155 133 we have discussed before may serve as effective biomarkers in NAFLD. Moreover, our group also isolated plasma exosomes from mice with MCD-induced NASH and found that the expression levels of miR-144-3p, miR-23b-3p, and miR-126a-5p were notably upregulated; however, the roles of these miRNAs in NASH remain unknown. The lack of a unified standard for the isolation of EVs and limited sequencing technology capabilities constrain the development of EVs as biomarkers in NAFLD. Additionally, the validity and usefulness of utilizing circulating exosomal miRNAs as biomarkers in NAFLD require further investigation.

Given that miRNAs can regulate the progression of NAFLD *via* multiple target genes, treatment based on the relevant miRNAs has attracted increasing research interest. Many groups have already demonstrated the validity of miRNA-based treatment in animal models of NAFLD. For instance, Lin et al. reported that a miR-192 mimic delivered by lentivirus alleviated hepatic steatosis in mice 54. Most studies exploring the function of specific miRNAs have used miRNA mimics or anti-miRNA delivered by viruses or transgenic mice; however, these methods are mostly not appropriate for use in a clinical setting. This highlights the need to find an appropriate miRNA delivery system. EVs are stable in circulating blood and can encapsulate many molecules, and packaging miRNAs (siRNAs or drugs) into EVs represents a promising strategy for the treatment of NAFLD. Li et al. packaged miR-199a-5p/anti-miR-199a-5p into exosomes and injected them into mice, resulting in aggravated/attenuated hepatic steatosis, respectively 134. Additionally, He et al. reported the delivery of a miR-146b mimic to hepatocytes through lactosylated PDMAEMA nanoparticles 138. However, the efficiency and safety of these targeted delivery systems remain to be determined. Meanwhile, because they have multiple targets and also target multiple cell types, the off-target effects of miRNA-based therapy must also be considered.

Due to the limitations of research techniques, our understanding of the miRNAs involved in the pathogenesis of NAFLD remains incomplete. Moreover, we could not elaborate on each miRNA that participates in NAFLD. However, with the development of bioinformatics and the progress of animal and clinical research, an increasing number of miRNAs involved in the occurrence and development of NAFLD will be identified, and these miRNAs might offer guidance for the noninvasive diagnosis and treatment of NAFLD.

## Figures and Tables

**Table 1:** MiRNAs that regulate lipid metabolism in nonalcoholic fatty liver disease (NAFLD).

MiRNA	Circulation level	Liver expression	Function*	Identified targets
miR-21	↑^10,11^	↑^9,15^	↑	FOXA2, FOXO1, HNF4α, STAT3, INSIG2,^12^ LRP6,^13^ Hbp1,^15^ PPARα^16,17^
miR-122	↑^22,38^	↑^22^↓^23^	↓^25,28^↑^27^	Agpat1, Dgat1,^25^ CPEB1,^28^ Sirt1^29^
miR-34a	↑^10,11^	↑^32,30^	↑	Sirt1,^30,31,36^ HNF4α,^32^ PPARα^30^
miR-27a	↓^39^	↑^40^	↓^40^↑^41^	FAS, SCD1,^40^ Nrf2^41^
miR-29a	↓^42^	↓^43^in fibrosis	↓	HMGCR,^44^ DNMT3b,^45^ CD36,^43^ GSK3β^46^
miR-132	unknown	↑^47^	↑	FoxO3, PTEN, Sirt1, CYP2E1, P300^47^
miR-125b	↑^22^	↑^48^	↓	FAS^49^
miR-155	unknown	↑^50,51^	↓	Ces3/TGH,^50^ LXα^52^
miR-192	↑^11,22^	↓^53^	↓	SREBF1,^54^ SCD1^53^
miR-378	unknown	↑^55,56^	↑	Nrf1^56^

* ↑: aggravating hepatic steatosis; ↓: alleviating hepatic steatosis.

**Table 2:** MiRNAs that regulate insulin resistance in nonalcoholic fatty liver disease (NAFLD).

MiRNA	Circulation level	Liver expression	Function*	Identified targets
miR-152	unknown	↓^60^	↓	PTEN^60^
miR-499-5p	unknown	↓^61^	↓	PTEN^61^
miR-26a	unknown	↓^62^	↓	GSK3β, PKCδ, PKCθ, ACSL3, ACSL4, PCK1, TCF7L2^62^
miR-497	unknown	↑^63^	↑	InsR^63^
miR-15b	↑^69^	↑^64,69^	↑	InsR^64^
miR-30b	↑^65^	↑^65^	↑	SERCA2b^65^
miR-206	unknown	↓^66^	↓	PTPN1^66^
miR-190b	unknown	↑^67^	↑	IGF-1, ADAMTS9^67^

* ↑: aggravating insulin resistance; ↓: alleviating insulin resistance.

**Table 3:** MiRNAs that regulate oxidative stress and endoplasmic reticulum (ER) stress in nonalcoholic fatty liver disease (NAFLD).

MiRNA	Circulation level	Liver expression	Function*	Identified targets(references)
miR-26a	unknown	↓^75^	↓	EIF2α^75^
miR-421	unknown	↑^76^	↑	Sirt3^76^
miR-27a	↓^39^	↑^41^	↑	Nrf2^41^
miR-136	unknown	↑^77^	↑	MEG3^77^
miR-96-5p	unknown	↓^78^	↓	p66shc^78^

* ↑: promoting oxidative stress/ER stress; ↓: improving oxidative stress/ER stress.

**Table 4:** MiRNAs that regulate hepatocyte apoptosis in nonalcoholic fatty liver disease (NAFLD).

MiRNA	Circulation level	Liver expression	Function*	Identified targets(references)
miR-223	↑^88^	↑^89^	↑?	IGF-1R^81^
miR-24	unknown	↑^90^	↓?	BIM^82^
miR-21	↑^10,11^	↑^9,15^	↓?	PDCD4^83^
miR-34a	↑^10,11^	↑^32,30^	↑	Sirt1^84-86^
miR-296	unknown	↓^87^	↓	PUMA^87^

* ↑: promoting apoptosis; ↓: suppressing apoptosis.

**Table 5:** MiRNAs that regulate hepatic inflammation in nonalcoholic fatty liver disease (NAFLD).

MiRNA	Circulation level	Liver expression	Function*	Identified targets(references)
miR-194	unknown	↑^100^	↑^100^↓^101^	FXR/Nr1h4,^100^ TRAF6^101^
miR-223	↑^88^	↑^89^	↓	Cxcl10, TAZ,^89,88^ NLRP3,^88,105^ Igf1r^88^
miR-192-5p	↑^102^	↑^102^	↑	Rictor^102^
miR-378	unknown	↑^103^	↑	Prkag2^103^
miR-125b	↑^22^	↑^48^	↑	TNFAIP3^48^
miR-451	unknown	↓^104^	↓	Cab39^104^

* ↑: promoting inflammation; ↓: suppressing inflammation.

**Table 6:** MiRNAs that regulate liver fibrosis in nonalcoholic fatty liver disease (NAFLD).

MiRNA	Circulation level	Liver expression	Function*	Identified targets(references)
miR-21	↑^10,11^	↑^9,15^	↑	SMAD7,^111^ PPARα^16^
miR-122	↑^22,38^	↓^114^	↓	HIF-1α, vimentin, MAP3K3^114^
miR-146a-5p	unknown	↓^115^	↓	Wnt1, Wnt5a^115^
miR-214	unknown	unknown	↑?	Sufu,^118^ Mig-6^119^
miR-130a-3p	unknown	↓^120^	↓	TGFBR1, TGFBR2^120^
miR-221-3p	unknown	↑^121^	↑?	GNAI2^121^
miR-223	↑^88^	↑^89^	↓	Nlrp3, Cxcl10, TAZ, Igf1r^88^
miR-29a	↓^42^	↓^43^	↓	CD36^43^

## Abbreviations

NAFLD: nonalcoholic fatty liver disease
T2DM: type 2 diabetes mellitus
NASH: nonalcoholic steatohepatitis
HCC: hepatocellular carcinoma
HSC: hepatic stellate cell
miRNAs: microRNAs
EVs: extracellular vesicles
DNL: *de novo* lipogenesis
FAO: fatty acid oxidation
VLDL: very-low-density lipoprotein
TG: triglyceride
ER: endoplasmic reticulum
NAHCC: NAFLD-related HCC
FFAs: free fatty acids
IR: insulin resistance
ROS: reactive oxygen species
UPR: unfolded protein response
JNK: c-Jun N-terminal kinase
DCA: deoxycholic acid
TAZ: transcriptional coactivator with PDZ-binding motif
MFs: fibrogenic myofibroblasts
ALD: alcoholic liver disease
